# The Role of Autophagy in HIV Infection and Immunological Recovery of ART-Treated PLWH

**DOI:** 10.3390/v17070884

**Published:** 2025-06-23

**Authors:** Mayara Sabino Leite de Oliveira Duarte, Wlisses Henrique Veloso de Carvalho-Silva, Rafael Lima Guimarães

**Affiliations:** 1 Department of Genetics, Federal University of Pernambuco (UFPE), Recife 50670-901, Pernambuco, Brazil; mayara.sabino@ufpe.br; 2 Keizo Asami Institute (iLIKA), Federal University of Pernambuco (UFPE), Recife 50670-901, Pernambuco, Brazil; 3 Department of Immunology, Aggeu Magalhães Institute (IAM/FIOCRUZ), Recife 50670-420, Pernambuco, Brazil; wlissesveloso@gmail.com; 4 Life Sciences Nucleus, Agreste Academic Center (CAA), Federal University of Pernambuco (UFPE), Caruaru 55014-900, Pernambuco, Brazil

**Keywords:** antiretroviral therapy, immunological non-responders, CD4+ T lymphocytes, programmed cell death, pyroptosis

## Abstract

Human immunodeficiency virus (HIV) is responsible for acquired immunodeficiency syndrome (AIDS), a condition characterized by the depletion of CD4+ T lymphocytes, which predisposes individuals to opportunistic infections and, ultimately, death. Although antiretroviral therapy (ART) has substantially improved clinical outcomes, certain limitations persist. Notably, 15–30% of individuals undergoing ART achieve viral suppression but fail to restore adequate CD4+ T cell counts, being defined as immunological non-responders (INR) and remaining at increased risk of disease progression to AIDS. The impaired immune recovery in INRs is attributed to insufficient production and/or excessive destruction of CD4+ T lymphocytes, which can be modulated by autophagy process. This evolutionarily conserved mechanism is fundamental to lymphocyte development and activation as well as to programmed cell death pathways such as apoptosis, necroptosis, ferroptosis, and pyroptosis. These pathways are essential for understanding the impaired immune reconstitution observed in people living with HIV, whose inability to maintain immune homeostasis contributes to accelerated disease progression. This review explores the interplay between autophagy, HIV, and cell death mechanisms, highlighting its relevance in immunological recovery under ART and its potential as a therapeutic target.

## 1. Introduction

Human immunodeficiency virus (HIV) is a retrovirus from the Retroviridae family responsible for acquired immunodeficiency syndrome (AIDS), characterized by the severe depletion of CD4+ T lymphocytes. If left uncontrolled, it can lead to an increase in opportunistic infections, potentially resulting in death [1,2]. Since the implementation of antiretroviral therapy (ART), the number of AIDS-related deaths has significantly decreased, with a reduction of up to 69% since 2004 [3]. Despite considerable advancements in ART, certain limitations have persisted. Among these, 15–30% of people living with HIV (PLWH) undergoing ART achieve viral suppression but fail to recover adequate CD4+ T-cell counts. These individuals are classified as immunological non-responders (INR) and are at a higher risk of rapidly progressing to AIDS and HIV-related complications [4,5]. The inability to develop an effective immune response in INR during ART is associated with two main mechanisms: the insufficient production of CD4+ T lymphocytes and/or the exacerbated destruction of these cells [6,7]. The molecular mechanisms governing the pathogenesis of HIV infection in INR remain under investigation. However, autophagy has emerged as a key factor in the dynamics of this interaction, playing significant roles in viral replication, immune response regulation, and disease progression [8,9,10].

Autophagy is a lysosomal degradation process that is essential for innate and adaptive immunity, playing a crucial role in the control of viral infections, including HIV [11]. Recent studies have demonstrated physical interaction between HIV and autophagy pathway proteins, involving at least 20 genes. For instance, the HIV Nef protein interacts with BECLIN-1, functioning as an anti-autophagic factor that not only inhibits autophagy but also promotes viral replication and dissemination [12]. Another study on CD4+ T lymphocytes revealed that shortly after viral entry, the protein (Vpr) rapidly regulates the autophagy process and attenuates the levels of LC3 and BECLIN-1 [13].

Autophagy is indispensable for maintaining cellular homeostasis and for the proper development and function of lymphocytes. Furthermore, it contributes significantly to the regulation of different cell death forms, such as apoptosis, necroptosis, ferroptosis, and pyroptosis [14,15]. HIV infection leads to the dysregulation of iron homeostasis and reduced autophagy, stimulating ferroptosis-induced cell death [16]. Additionally, autophagy regulates necroptosis by negatively modulating the interaction between BECLIN-1 and RIPK3, thereby contributing to immune system depletion [10,17,18]. Apoptosis is another form of autophagy-associated cell death. The degradation of the Fas ligand inhibitor by autophagy can trigger this process [19], Conversely, the interaction of BECLIN-1 with PINK1 and BCL-2 promotes the formation of autophagosomes, demonstrating an anti-apoptotic effect [20]. Autophagy can also coordinate pyroptosis. Studies conducted in patients with HIV on lymphoid tissues have indicated that 95% of infected cells die by pyroptosis in these tissues [21,22]. Once activated, pyroptosis results in lytic cell death and the release of the inflammatory cytokines IL-1β and IL-18, ultimately reducing autophagic activity [23,24,25].

Autophagy emerges as a key process in the interaction with HIV, as it is fundamental in the regulation of T-lymphocyte formation and activation as well as in cell death mechanisms [8,10,23]. Together, autophagy and cell death interactions form a complex network that drives HIV pathogenesis and host impaired immune response. A deeper understanding of these mechanisms is essential to comprehend the immune dysfunction observed in PLWH, whose inability to establish immune homeostasis accelerates the progression of infection to AIDS [8,23].

Through this literature review, we provide an overview of the autophagy–HIV–cell death interaction, highlighting the central role of autophagy by exploring its function in the context of HIV infection, its underlying mechanisms, and its influence on the immunological recovery of PLWH under ART. By addressing these aspects, the review seeks to address existing knowledge gaps and stimulate new lines of investigation, thereby providing a basis for future research and contributing significantly to the advancement of this field. Ultimately, it may support the development of novel therapeutic or immunological strategies for INR.

## 2. General Process of Autophagy

Autophagy is a catabolic process characterized by the formation of a double-membrane structure that allows the degradation and recycling of cellular substrates, including microorganisms and damaged proteins [24]. This process involves cellular homeostasis by providing an autonomous source of energy and amino acids. Autophagy can be activated by various stimuli, such as nutrient deprivation, reactive oxygen species (ROS), cell death, and infectious agents, including HIV [25,26].

Different forms of autophagy have been identified based on the nature of the cargo and the mechanisms by which it is delivered to lysosomes. Currently, three major forms have been described: macroautophagy, chaperone-mediated autophagy (CMA), and microautophagy [4,27]. Microautophagy is characterized by the direct capture of cytoplasmic components via invagination of the lysosomal membrane or, in the case of endosomal microautophagy, the membrane of late endosomes [28]. In contrast, CMA involves the elective recognition of cytosolic proteins by the heat-shock cognate protein Hsc70, which facilitates their direct translocation across the lysosomal membrane into the lumen for degradation [27,29].

Macroautophagy, commonly referred to simply as autophagy, is characterized by the sequestration of cytoplasmic components, including organelles, within double-membrane vesicles known as autophagosome [12,30,31]. These vesicles subsequently fuse with lysosomes, where their contents are degraded and recycled. In addition to its general function, macroautophagy can occur in a selective manner, targeting specific substrates [32,33]. Several selective forms have been identified, including mitophagy (targeting mitochondria), reticulophagy (endoplasmic reticulum), lysophagy (lysosomes), aggrephagy (protein aggregates), pexophagy (peroxisomes), lipophagy (lipid droplets), ribophagy (ribosomes), ferritinophagy (iron-storage proteins), and xenophagy (intracellular pathogens) [26,32].

## 3. Autophagy Regulation Mechanism

Autophagy is regulated by more than thirty autophagy-related genes (ATGs), which encode proteins that can be functionally categorized into four main groups for clarity: (1) the ULK1 (Unc-like kinases) complex and its regulatory components, whose assembly is independent of the cell’s nutritional status but whose activity is modulated through various phosphorylation events; (2) the Class III phosphatidylinositol 3-kinase (PI3K) complex I, also known as the VPS34 complex; (3) the conjugation systems responsible for recycling and processing ubiquitin-like proteins involved in autophagosome formation; and (4) the ATG8/LC3 conjugation complex, essential for membrane expansion and cargo recruitment [32,34,35].

### 3.1. ULK Complex

**Figure 1:**
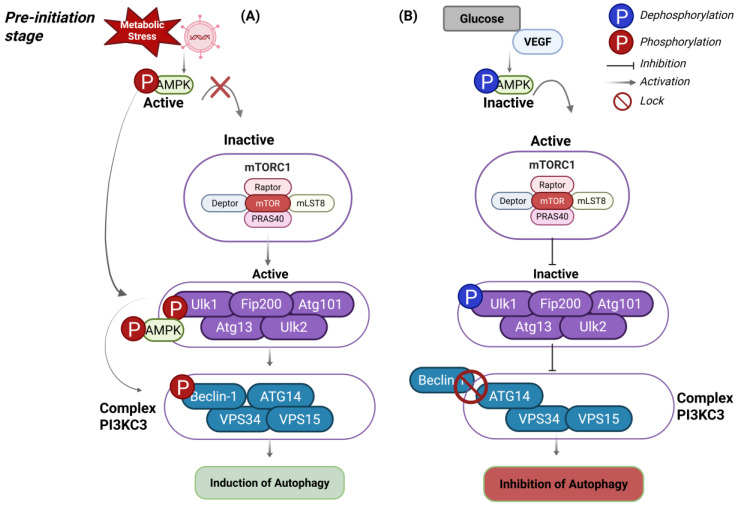
Induction and inhibition mechanisms of autophagy process. (**A**) Stimuli such as nutritional stress and pathogenic exposure activate the AMPK sensor, which in turn phosphorylates the ULK protein, thereby activating the ULK1 complex. The phosphorylated complex promotes the activation of BECLIN-1, which, under basal conditions, is sequestered by its interaction with the anti-apoptotic protein BCL-2. Upon phosphorylation, BECLIN-1 dissociates from BCL-2 and initiates the activation of the second complex, VPS34 (also known as PI3KC3). (**B**) Conversely, in the presence of increased glucose levels and growth factors stimulation, the mTORC complex is activated. As a result, it inhibits autophagy by preventing ULK1 complex activation and blocking the interaction between BECLIN-1 and VPS34.

In response to cellular stressors such as nutrient deprivation, elevated temperature, and pathogenic infections, the energy sensor AMPK (5′ AMP-activated protein kinase) is activated [36]. AMPK consists of three subunits (α, β, and γ), and upon activation, it phosphorylates the ULK1 complex at serine residues 317 and 777, thereby activating ULK1 and initiating autophagy though AMPK–ULK1 interaction (Figure 1A) [37]. Conversely, under conditions of nutrient sufficiency and growth factor stimulation, the mTORC1 (mechanistic target of rapamycin complex 1) interacts with the ULK1 complex and inhibits autophagy by promoting ULK1 dephosphorylation at serine 757 (Figure 1B) [26].

### 3.2. VPS34 Complex

VPS34 is a crucial protein involved in regulating cellular stress through its roles in autophagy and endocytosis. Beyond autophagy, VPS34 acts as a fundamental modulator of multiple pathophysiological processes. For instance, mice carrying homozygous mutations in the kinase domain of VPS34 exhibit embryonic lethality, highlighting its essential role in embryogenesis [32,38,39]. The VPS34 complex becomes selectively involved in autophagy when it is associated with BECLIN-1 and other regulatory proteins. VPS34 utilizes phosphatidylinositol (PI) as a substrate to generate phosphatidylinositol 3-phosphate (PI3P), which is essential for phagophore elongation and the recruitment of ATG proteins to the phagophore membrane [40].

### 3.3. ATG5–ATG12 Conjugation

Two ubiquitin-like conjugation systems are essential for autophagy: the ATG5–ATG12 conjugation and the LC3 processing pathway. In the first system, ATG7 acts as an E1-like activating enzyme that activates ATG12 in an ATP-dependent manner. ATG12 is then transferred by ATG10, an E2-like carrier protein, facilitating its covalent binding to lysine 130 of ATG5 [32,35]. Conjugated ATG5–ATG12 associates with ATG16L dimers, forming a multimeric ATG5–ATG12–ATG16L complex. This complex acts as an E3-like enzyme, catalyzing the lipidation of LC3-II and its recruitment to the phagophore membrane [38,41]. Notably, the ATG5–ATG12 conjugation occurs independently of autophagy induction. During autophagosome maturation, the complex dissociates from the membrane, rendering it a relatively weak marker of autophagy [41,42,43].

### 3.4. The ATG8/LC3 Conjugation Complex

The second ubiquitin-like system crucial for autophagosome formation involves the processing of microtubule-associated protein light chain 3 (LC3). LC3/ATG8 are ubiquitin-like proteins that must be covalently conjugated to phosphatidylethanolamine (PE) to perform their function [41,44]. While yeast expresses a single ATG8 protein, the mammalian ATG8 family includes at least six orthologs classified into two subfamilies. The human LC3 gene family consists of three members: LC3-I, LC3-II, and LC3-III, whereas the GABARAP subfamily comprises GABARAP, GABARAP-L1, and GABARAP-L2 [45].

Both LC3-I and LC3-II are differentially expressed across normal tissues. Conversely, LC3-III appears to be poorly expressed or even absent in most normal tissues [46,47,48,49]. Regarding characterization, LC3-II is the most extensively studied and is widely recognized as a marker of autophagosomes, serving as the canonical representative of the LC3 subfamily [50]. The non-canonical ubiquitin-like conjugation cascade described above—comprising E1 (ATG7), E2 (ATG3 and ATG10), and the E3-like ATG12–ATG5–ATG16 complex—is essential for the covalent conjugation of LC3 to PE, an important step for its incorporation into the expanding phagophore [51].

## 4. Molecular Machinery of Autophagosome Formation

Autophagy is initiated through a pre-initiation step, that in mammals is coordinated by the ULK1 complex downstream of multiple signaling pathways involved in nutrient detection and metabolic regulation [35,52,53]. Upon activation, ULK1 stimulates VPS34 complex, triggering the phagophore (isolation membrane) formation, which originates from lipid bilayers derived from the endoplasmic reticulum (ER), trans-Golgi network, and/or endosomes [35,41]. Phagophore elongation is driven by two ubiquitin-like conjugation systems: ATG12–ATG5 and LC3–PE. In the first system, ATG12 is conjugated to ATG5 in a process mediated by ATG7 (E1-like enzyme) and ATG10 (E2-like enzyme). Subsequently, ATG16L associates with the ATG12–ATG5 complex, facilitating the lipidation of LC3. Pro-LC3 is initially cleaved at its C-terminus by the protease ATG4 to generate cytosolic LC3-I, which is then conjugated to PE, producing the membrane-bound form LC3-II. [29,54]. Once the autophagosomes sequester and enclose their cargo, they fuse with lysosomes to form autolysosomes, where the internal content is degraded [32]. This fusion and maturation process is mediated by members of the Rab and SNARE (soluble N-ethylmaleimide-sensitive factor attachment protein receptor) protein families [27,55].

### 4.1. Initiation

The initiation phase of autophagy is stimulated by the assembly of a pre-initiation complex composed of five proteins: ATG101, ATG13, FIP200, and the kinase proteins ULK1 and ULK2. Among these, ATG101, ATG13, and FIP200 interact with the kinase family, while ULK1 and ULK2 play central regulatory roles. For instance, activation of the cellular stress sensor AMPK leads to the phosphorylation and subsequent activation of the ULK1 complex [23,35,53]. Once the ULK complex is phosphorylated, it can initiate autophagy through two main mechanisms: (1) directly phosphorylating and activating the Class III phosphatidylinositol 3-kinase complex I (PI3KC3), composed of BECLIN-1, ATG14, vacuolar protein sorting kinase 34 (VPS34), and the membrane-anchoring protein VPS15 (Figure 1A) [32,56], or (2) indirectly facilitating via the phosphorylation of AMBRA1, a regulatory protein [57,58].

### 4.2. Elongation Maturation and Lysosomal Degradation

**Figure 2:**
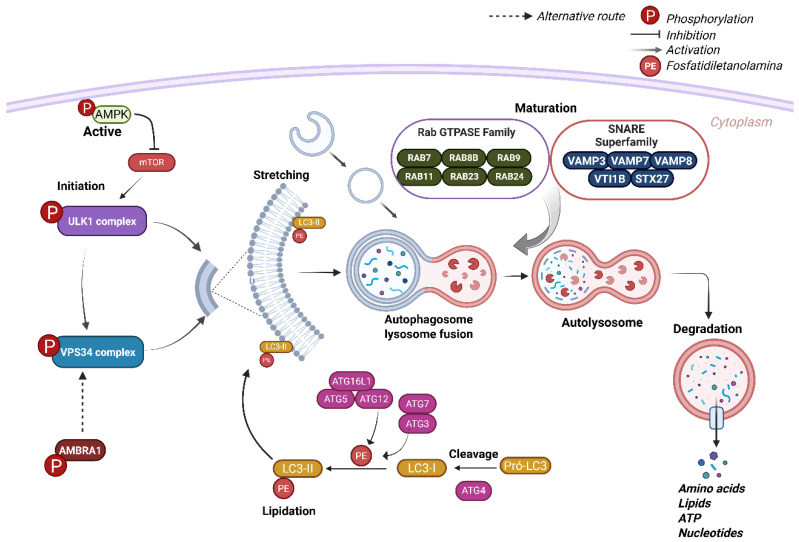
Molecular machinery of autophagosome formation. The general mechanism of autophagy begins with the activation of AMPK, which phosphorylates the ULK1 complex and consequently activates the VPS34 complex. The VPS34 complex can also be activated directly by AMBRA1. Once the VPS34 complex is activated, the nascent phagophore is formed, and the LC3-I protein is cleaved by ATG4 and lipidated by the conjugates ATG7, ATG3, ATG5, ATG12, and ATG16L1. In its lipidated form, LC3-II along with ATGs binds to the phagophore to form the autophagosome.

The elongation stage is marked by the progressive expansion of the autophagic membrane, a process tightly regulated by ATG5–ATG12–ATG16 complex and the LC3 processing [23,59]. Under basal conditions, LC3 is found in cytosolic forms, often referred as pro-LC3. Upon autophagy induction, LC3 is recruited to the growing phagophore, where the cysteine protease ATG4 cleaves its C-terminal regions, exposing a glycine residue and converting it to LC3-I [60]. This enables covalent attachment of PE to LC3-I, modifying it into its lipidated form, LC3-II. This lipidation is mediated sequentially by ATG7, ATG3, and the ATG5–ATG12–ATG16L1 complex (Figure 2) [54].

After autophagosome formation, maturation and lysosomal degradation begin, facilitated by ATG9 and ATG18, which recycle autophagic components. This stage is regulated by various proteins, especially members of the Rab GTPase family (e.g., RAB7, RAB8B, RAB9, RAB11, RAB23, and RAB24) [59,60] and SNARE family—such as VAMP3, VAMP7, VAMP8, VTI1B, and STX17 [61]. Fusion allows lysosomal hydrolases to break down autophagosomal contents, which are then recycled to maintain metabolic homeostasis [23] (Figure 2).

## 5. Autophagy in Innate and Adaptive Immune Responses

Autophagy is crucial to immunity and is regulated by pattern recognition receptors (PRRs), such as TLRs and NODs, upon detecting pathogen-associated molecular patterns (PAMPs) [11]. For instance, TLR7 induces the conversion of LC3-I to LC3-II after exposure to HIV, resulting in viral degradation [14]. Yue-Ming showed that autophagy controls monocyte proliferation and inhibits differentiation into macrophages [62]. In 2007, pioneering work led by Douglas R. Green reported that autophagic proteins such as BECLIN-1, LC3, ATG5, and ATG7 can be recruited to autophagosomes in macrophages upon TLR activation. This enhances acidification and pathogen destruction, a process known as LC3-associated phagocytosis (LAP) [63,64].

PAMP recognition by macrophages also activates inflammasomes—cytosolic complexes that drive inflammation and pyroptosis [14]. Evidence indicates that inflammasome activation induced by endotoxins in mice is regulated by the autophagic protein ATG16L1. Without ATG16L1, the ATG12–ATG5 complex fails to recruit properly, disrupting LC3-II conjugation, autophagosome formation, and degradation capacity [65]. LAP is also crucial in adaptive immunity by enabling pathogen degradation and subsequent presentation to CD4+ T cells via MHC class II (MHC-II) molecules [63]. Romão [66] found that LC3-II preserves antigens in macrophages and dendritic cells, sustaining MHC-II presentation [66].

In antigen-presenting cells (APCs), extracellular antigens are processed in autophagosomes into immunogenic peptides for CD4+ T-cell presentation. In Crohn’s disease, dendritic cells with ATG16L1 variants show impaired autophagy and reduced MHC-II presentation [67]. Additionally, rapamycin, an autophagy inducer and mTOR inhibitor, enhances mycobacterial antigen presentation and CD4+ T-cell activation. These findings highlight autophagy’s dual role in both pathogen degradation and efficient activation of the adaptive immune response [68].

## 6. Autophagy and HIV

The HIV genome is composed by three major genes (*gag*, *pol*, and *env*), along with regulatory (*tat*, *rev*, and *nef*) and accessory (*vif*, *vpr*, and *vpu*—HIV-1 or *vpx*—HIV-2) genes [12,36,69]. Together, these genes encode proteins that orchestrate viral pathogenesis by subverting the host immune response. The HIV-1 protein Vpu, for example, interacts with the autophagic isoform LC3-III and impairs the degradation of viral particles by neutralizing bone marrow stromal cell antigen 2 (BST2), a host protein that normally restricts viral release and propagation [70].

During viral entry into the host cell, the HIV Nef protein binds to BECLIN-1, preventing the fusion of autophagosomes with lysosomes and suppressing autophagic processes [71]. In the early stages of autophagy, Kyey et al. demonstrated that Gag-p17 interacts with LC3II, promoting HIV production [71]. In another study, an autophagy-inducing peptide named Tat-Beclin-1 (TB1), a fusion of the HIV Tat cell-penetrating peptide with a fragment of BECLIN-1, was evaluated in the human colon carcinoma cell line HCT116. TB1 has been described as a potent autophagy inducer and antiviral agent against replicative viruses such as HIV and chikungunya. Furthermore, it enhances the transduction efficiency of human CD34+ hematopoietic stem/progenitor cells, highlighting the contribution of autophagy to effective immune responses and to the control of clinically significant viral infections [70].

Studies have shown that the autophagic protein ATG10S interacts with IFNL2 (interferon lambda 2), promoting the formation of autolysosomes and resulting in the degradation of viral proteins, including reverse transcriptase of HIV [72,73,74]. In addition, HIV envelope glycoprotein complex (Env) has been reported to induce autophagy and subsequent cell death in uninfected CD4+ T lymphocytes after prolonged virus–cell interactions [75]. The Nef protein interacts with the autophagy maturation factor BECLIN-1, thereby inhibiting autophagy and protecting HIV from degradation. In macrophages, Tat interacts with LC3-II, using the nascent autophagy double membrane as a scaffold to enhance viral replication and inhibit autophagy, respectively, further facilitating HIV survival and propagation [71,76].

The first interaction between HIV envelope complex (Env) and autophagy was reported in 2006, when it was demonstrated that cells transfected with Env induced autophagy and BECLIN-1 accumulation in uninfected CD4+ T lymphocytes through activation of the CXCR4 receptor. This interaction leads to apoptosis, suggesting that HIV-mediated autophagic death of CD4+ T lymphocytes may play a significant role in the development of immunodeficiency [75,77]. A study evaluating CD4+ T lymphocytes showed that shortly after the virus entry, the viral protein Vpr rapidly regulates the autophagy process by reducing LC3II and BECLIN-1 levels [13]. Research conducted with PLWH classified as long-term non-progressors (LTNPs) and elite controllers (LTNP-ECs) demonstrated that these phenotypes exhibited elevated levels of autophagy-related protein expression and a higher number of autophagic vesicles in their PBMCs compared to normal progressors [78,79].

Another study also highlighted that rapamycin treatment in PBMC from LTNP-EC individuals stimulated autophagy and reduced viral production, demonstrating that the autophagy mechanism is not only essential for viral replication cycle but also plays a key role in the CD4+ T-lymphocytes depletion and, consequently, in the immune reconstitution of these patients [36]. Moreover, it has been shown that the Vif protein, essential during the final stages of HIV replication, can interact with LC3-II, resulting in the inhibition of autophagy [80].

**Figure 3:**
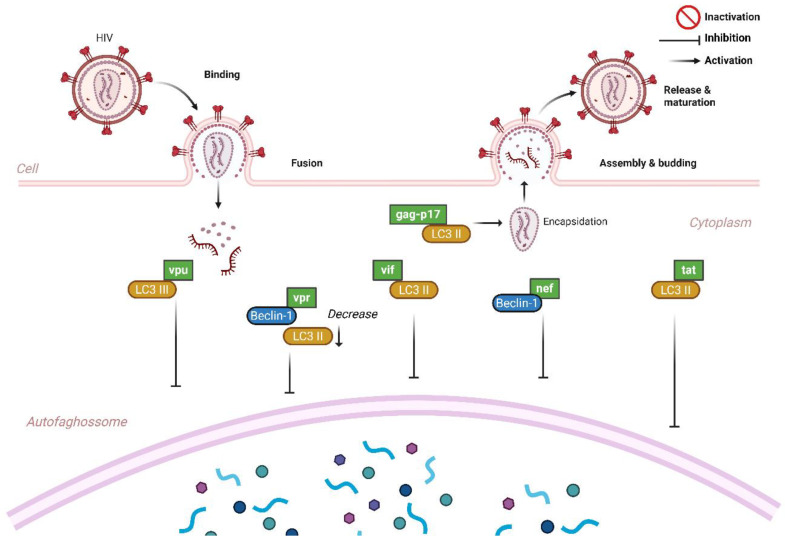
Interaction between HIV and autophagy process during viral replication. During infection, HIV proteins interact directly with autophagy components to inhibit viral degradation. Vif and Gag-p17 suppress autophagosome formation by interacting with LC3-II and by Vpu interacting with LC3-III. Shortly after viral entry, the Vpr protein regulates autophagy by attenuating LC3-II and BECLIN-1 levels. The Gag protein manipulates LC3-II to promote the formation of a nascent phagophore, providing a protective niche for viral replication. The Nef protein binds to the maturation factor BECLIN-1, further inhibiting autophagy and protecting HIV from degradation.

Autophagy plays contrasting roles in HIV infection. Early in the infection, the Tat protein has the potential to induce autophagy by inhibiting the mTOR pathway and activating AMPK. When activated by Tat, AMPK promotes a significant increase in autophagy, thereby enhancing the availability of essential metabolites required for efficient HIV replication [81]. Although the precise role of the HIV–autophagy interaction remains complex and not fully understood, the link between autophagy and both HIV pathogenesis and the outcome of the host immune response is well established (Figure 3). In addition, Table 1 summarizes some studies emphasizing the interaction between HIV proteins and autophagy pathway.

## 7. Autophagy and Immunological Recovery in PLWH Under ART

CD4+ T lymphocytes are fundamental cells of the immune system and essential for defense against HIV infection. Studies have indicated that robust CD4+ T-lymphocyte responses against HIV are crucial for reducing viremia and gradually restoring CD4+ T-cell counts [85,86]. However, according to the literature, despite achieving complete viral suppression, 15–30% of individuals who initiate ART experience significant difficulties in recovering CD4+ T-cell levels, leading to a higher risk of HIV-related complications and mortality. These individuals are classified as immunological non-responders (INR) [5,87]. Considering a multifactorial condition, several factors have been associated with INR status, such as advanced age, sex, co-infections during treatment, CD4+ T-cell count at baseline, and genetic alterations. Furthermore, previous studies have highlighted two main mechanisms underlying this phenomenon: insufficient production and excessive destruction of CD4+ T lymphocytes [6,88,89,90].

The identification and study of PLWH defined as EC have provided important insights into cellular processes related to disease progression, including the role of autophagy [8]. As previously mentioned, autophagy is a fundamental process in the immune responses against HIV infection, contributing to the regulation of T-lymphocyte maturation and activation as well as to mechanisms of cell death. Thus, the following subsections explore how autophagy may modulate the two main mechanisms of incomplete immune reconstitution in PLWH undergoing ART, offering new perspectives for understanding and addressing the limitations in INRs.

### 7.1. Antiretrovirals and Autophagy

The World Health Organization (WHO) has raised concerns about the increasing resistance of HIV to ART, especially involving nucleoside reverse-transcriptase inhibitors (NRTIs). Although many countries have adopted dolutegravir (DTG) as the preferred first- and second-line treatment, low- and middle-income nations continue to face barriers to access and often rely on alternative drug regimens [91]. Antiretroviral drugs can cause a range of adverse effects in PLWH, including hepatotoxicity and cardiotoxicity. These toxic effects may be partially attributed to autophagy dysregulation in different cells, tissues, and organs, potentially contributing to the immune dysfunction observed in these individuals [91,92].

Two studies have shown that an ART regimen consisting of two NRTI (nucleoside reverse transcriptase inhibitors) plus an INI (integrase inhibitors) inhibits autophagy in PLWH. One study evaluated the effects of tenofovir disoproxil fumarate (TDF), emtricitabine (FTC), and dolutegravir (DTG) on rat microglial cells, reporting abnormal lysosomal function and impaired autophagosome maturation after 24 h of treatment with the TDF + FTC + DTG combination [93]. Another study observed that the combination of TDF + FTC + DTG increased ROS by inhibiting autophagic flux in microglia in rats [94]. Another study demonstrated that a combination of tenofovir (TDF), FTC, and raltegravir (RAL) inhibits autophagy in human astrocytes by blocking autophagosome formation [95].

Although zidovudine (ZDV) is now less commonly used, and stavudine (d4T) has been commercially removed, both drugs were widely employed in earlier ART regimens. ZDV and d4T have been shown to inhibit autophagy in most examined cell types, including myoblasts, adipocytes, and fibroblasts, potentially contributing to adverse effects such as myopathy and lipodystrophy. Notably, patients treated with ZDV-based regimens exhibited impaired immune recovery compared with those on non-ZDV-based regimens. The authors suggested that ZDV-induced autophagy inhibition in T cells might reduce T-cell survival [96,97].

Lamivudine (3TC), still an important component of ART and widely used for post-exposure prophylaxis (PEP), has been evaluated in myocytes, adipocytes, and hepatocytes. However, it has shown no significant effect on mitochondria or autophagy in any of these cell types [97].

Atazanavir (ATV), often preferred in pregnant women and patients with first-line treatment failure [5,98], appears to exert a protective metabolic profile, as it enhances autophagosome formation in adipocytes and stimulates autophagy [99].

Collectively, these findings confirm that antiretrovirals modulate autophagy in drug- and cell-specific ways. These insights underscore the therapeutic relevance for targeting autophagy. For instance, drugs such as efavirenz (EFV) have been evaluated for their cytotoxic effects on cancer cells via autophagy modulation [100,101]. Additionally, d4T, explored in the context of Alzheimer’s disease, appears to enhance macrophage phagocytosis—an effect possibly mediated by autophagy [102] (Table 2).

### 7.2. The Role of Autophagy in the T-Lymphocytes Production

An effective response against HIV depends on the efficiency of CD4+ T-lymphocyte activity. Autophagy plays an essential role in antigen detection, presentation, survival, and maintenance of T lymphocytes [59,103]. A study conducted with embryonic stem cells in vitro, investigating the role of BECLIN-1 in lymphocyte development, revealed that targeted disruption of BECLIN-1 in mice resulted in early embryonic lethality before the establishment of a lymphoid system as well as a marked reduction in autophagic activity in these cells. These findings indicated that BECLIN-1 is fundamental for the preservation of lymphoid progenitor populations during early or undifferentiated stages [63,104].

Furthermore, the inactivation of ATG5 and ATG7 in T lymphocytes resulted in various intrinsic defects affecting both thymocytes and peripheral T cells. In thymocytes, reduced thymic cellularity and compromised cell survival were observed [105,106]. Similarly, peripheral T cells exhibited reduced numbers of both CD4+ and CD8+ T cells, alongside impaired survival, defective proliferative capacity, altered activation profiles, memory dysfunction, and compromised homeostatic expansion [104].

Another study using mice deficient in the autophagic protein ATG5 (ATG5-/-) demonstrated a substantial reduction in thymic cellularity as well as a significant decrease in the number of peripheral T and B lymphocytes. ATG5-/- CD8+ T lymphocytes exhibited a markedly increase rate of cell death. Similarly, ATG5 −/− CD4+ and CD8+ T cells showed impaired proliferative capacity in response to TCR stimulation [106] Additionally, a study evaluating the exposure of monocyte-derived dendritic cells (DCs) via LC3 to HIV showed that the virus induced mTOR activation, resulting in a significant reduction in CD4+ T-cell responses. Conversely, the induction of autophagy in DCs using rapamycin treatment resulted in a robust enhancement of CD4+ T-cell responses [107].

### 7.3. Cell Death Induced by Autophagy

**Figure 4:**
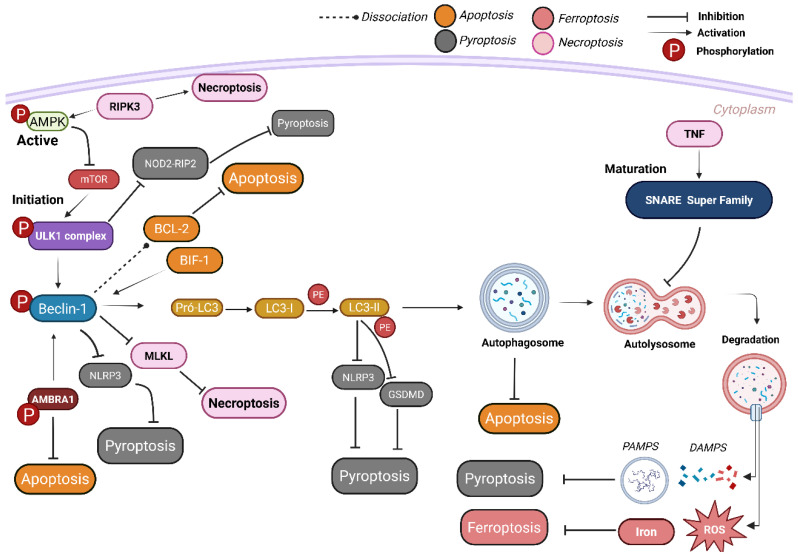
Modulation of autophagy in different cell death pathways. Ferroptosis (salmon-colored) is inhibited by the elimination of iron and oxidative stress via autophagy. Necroptosis (light pink) can be suppressed by the interaction of BECLIN-1 with RIPK1, inhibition of MLKL by BECLIN-1, and dysregulation of SNARE proteins by TNF; additionally, RIPK3 can directly associate with AMPK, inducing necrosome formation and reducing autophagy. Apoptosis (orange) can be inhibited through direct interaction between AMBRA1 and BECLIN-1. Bif-1 also interacts with BECLIN-1 and LC3-II, promoting autophagosome formation and preventing apoptosis. Another way to regulate apoptosis involves the activation and dissociation of the BECLIN-1/BCL-2 complex. Pyroptosis (gray) can be inhibited through multiple mechanisms, including inhibition of NOD2 by ULK-1, inhibition of NLRP3 by BECLIN-1 and LC3-I, inhibition of GSDM by LC3-I, and elimination of DAMPs and PAMPs. On the other hand, increased release of IL-β and IL-18 upon ATG16L1 knockout promotes pyroptosis.

It is currently known that HIV infection, beyond inducing traditional and widely studied forms of cell death such as apoptosis and pyroptosis, also promotes metabolic alterations that trigger additional forms of cell death, including ferroptosis and necroptosis (Figure 4) [10,18,25]. Dysregulation in iron homeostasis exacerbates HIV infection, and the concomitant use of ART may increase the risk of ferroptosis by intensifying ferritin autophagy at lysosomal level [16]. Ferroptosis is a regulated form of cell death characterized by the accumulation of iron-dependent lipid hydroperoxides and ROS, contributing to the loss of immune cells and damage to lymphoid tissues. In Xiao’s study (2022), a reduction was observed in recent thymic emigrants (RTEs), impaired cytotoxic function, increased inflammation, and elevated lipid peroxidation, all indicative of ferroptosis in CD4+ T lymphocytes from INRs [16]. Notably, it was observed that autophagy can reduce ferroptosis by removing oxidized lipids and regulating intracellular iron accumulation [40].

Necroptosis, a regulated form of cell death mediated by proteins such as RIPK1 (Receptor-Interacting Protein Kinase 1) and RIPK3 (Receptor-Interacting Protein Kinase 3), has also been implicated in HIV pathogenesis [108,109]. During HIV infection, gp41 induces necroptosis via RIPK1 activation, leading to the release of pro-inflammatory cytokines and chemokines and contributing to CD4+ T-cell depletion [110]. Necroptosis mediated by RIPK3 has been associated with AMPK activation, which promotes necrosome formation and induces cell death in both infected and uninfected cells, thereby contributing to immune system impairment [10,18]. A study investigated the induction of autophagy and necroptosis in mesenchymal stem cells (MSCs) using RUBCNL (Beclin 1-interacting protein with RUN and a cysteine-rich domain) as an inducer, and it was seen that RUBCNL expression, mediated by RIPK1, protected MSCs from both necroptosis and apoptosis, suggesting that the formation of the RUBCNL–RIPK1 complex may promote autophagy while concurrently suppressing necroptosis and apoptosis. BECLIN-1 also acts as a negative regulator of necroptosis by interacting with the necrosome complex, particularly MLKL, thereby preventing plasma membrane rupture and subsequent necroptotic cell death [17]. Additionally, it has been observed that necroptosis induced by tumor necrosis factor (TNF) impairs autophagolysosome degradation by disrupting the regulation of SNARE proteins [18,111].

Autophagy and apoptosis are intrinsically interconnected and regulated by effector proteins from different pathways. For example, autophagy can promote apoptosis by degrading inhibitors of the FasL (CD95/Apo-1), but it can also attenuate apoptosis by modulating the levels of Bcl-2 family proteins [112]. Under normal conditions, the interaction between autophagic proteins BECLIN-1 and Bcl-2 inhibits autophagy. Upon phosphorylation of BECLIN-1, this interaction is disrupted, leading to the induction of autophagy and the reduction in apoptosis-related cell death [19,113]. Another mechanism of apoptosis regulation involves the interaction between the pro-apoptotic protein Bax and the interaction factor Bif-1. Bif-1 forms a complex with BECLIN-1, promoting the activation of the Vps34 complex and LC3-II protein, thereby facilitating autophagosome formation. For example, during rabies virus (RABV) infection, Bif-1 is upregulated, leading to reduced apoptosis and RABV replication in neuronal cells [9,114]. Moreover, it has also been observed that the AMBRA1 protein, induced by paclitaxel, modulates apoptosis in cancer cells. The expression of AMBRA1 decreases during staurosporine-induced apoptosis through its interaction with BECLIN-1, demonstrating its role as a key regulator of apoptosis [115,116] (Figure 4).

### 7.4. Regulation of Autophagy in Pyroptosis

Since early investigations into the mechanisms underlying CD4+ T-lymphocyte depletion, apoptosis has been considered the primary form of cell death in PLWH. However, studies using human lymphoid tissues revealed that only approximately 5% of HIV-infected cells underwent apoptosis, whereas the remaining 95% died via pyroptosis [21,22,117]. Pyroptosis is a highly inflammatory cell death regulated by gasdermin (GSDM) family proteins and is triggered by signals such as PAMPs and DAMPs. This process results in lytic cell death and the release of pro-inflammatory cytokines IL-1β and IL-18, which have been shown to reduce autophagy activity [89,117,118].

In a study using ATG7 knockout mice, increased inflammasome activation, elevated levels of IL-1β and IL-18, and enhanced pyroptosis were observed in macrophages [118]. Furthermore, Chen et al. described that inflammatory myocardial injury demonstrated that autophagy plays a crucial role in degrading NLRP3 inflammasomes, thereby reducing inflammation and promoting the phenotypic shift of macrophages from a pro-inflammatory M1 profile to an anti-inflammatory M2 state [119]. Key genes involved in autophagy, such as ULK-1 and ATG9, have been shown to suppress the STING signal and inhibit inflammation and pyroptosis [120]. Hua et al.’s research evaluating chronic inflammation using exosomes derived from mesenchymal stem cells (huc-MSCs) demonstrated that these vesicles suppress neuroinflammation by inhibiting pyroptosis via GSDM regulation, increasing the expression of BECLIN-1 and LC3-II and promoting autophagy [121]. It was discovered by Wu et al. that ATG5 downregulation resulted in ROS production, triggering NLRP3 activation [122]. A recent study on Wistar rats showed that renal toxicity induced by the chemotherapy drug sunitinib (SUN) is associated with increased activation of NLRP3 inflammasomes and inflammatory mediators (IL-1β, END-1, and MCP-1), followed by a reduction in BECLIN-1 expression. Co-treatment with drugs such as secukinumab (SEC) and dapagliflozin (DAPA) reduced NLRP3-induced inflammation and stimulated autophagy via BECLIN-1 [123].

**Figure 5:**
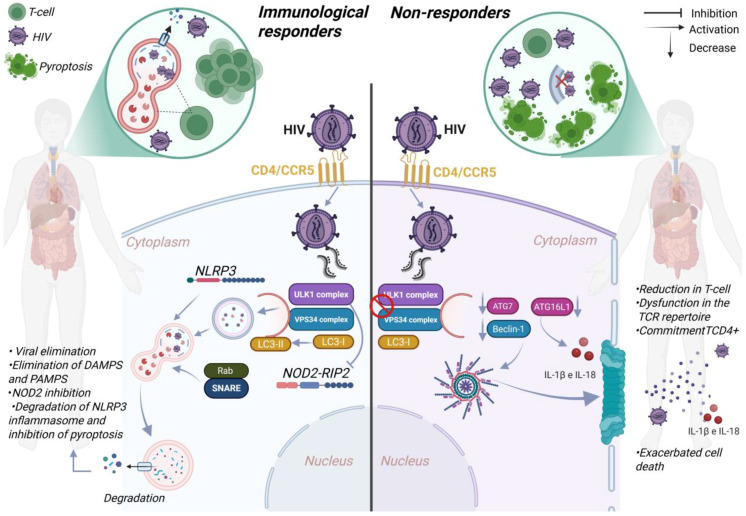
Autophagy may be related to the immune response in individuals living with HIV through its interaction with essential proteins in the pyroptotic pathway. In immunological responders (IR), autophagy acts by degrading PAMPs and DAMPs, reducing the release of the inflammatory cytokines IL-1β and IL-18, and consequently suppressing pyroptosis. Autophagy modulation can also occur through inactivation of NOD2-RIP2 by the ULK-1 complex, while NLRP3 inflammasomes can be degraded by autolysosomes, preventing pyroptotic cell death. Autophagy modulation can also occur through inactivation of NOD2-RIP2 by the ULK-1 complex, while NLRP3 inflammasomes can be degraded by autolysosomes, preventing pyroptotic cell death. And ULK-1 and ATG9 have been shown to suppress STING signal and inhibit inflammation and pyroptosis. Additionally, autophagy can degrade HIV by inhibiting viral replication and spread. In immunological non-responders (INR), ULK1 and VPS34 complexes are inhibited by HIV viral proteins, and the reduction in ATG7 and BECLIN-1 induces inflammasome formation and pyroptotic death. Suppression of ATG5 increases the NLRP3 inflammasome. ATG16 deficiency further intensifies the release of IL-1β and IL-18—pro-inflammatory cytokines and intracellular contents that recruit healthy CD4+ T cells, triggering an intense inflammatory process. Moreover, autophagy dysfunction can reduce the number of CD4+ T lymphocytes in the thymus and periphery, impair early functional capacity of CD4+ T cells in cytokine production, compromise the effectiveness of effector CD8+ T cells, and directly impact immune recovery in people living with HIV.

It was also observed that during *Streptococcus pneumoniae* infection, an increase in ULK1 was shown to reduce pyroptosis through the NOD2-RIP2 pathway in microglial cells [124]. The interaction between NLRP3 and the autophagic protein LC3II was highlighted by Luo (2023), who found that the phytohormone bergapten (BeG) inhibited NLRP3 activation by increasing LC3-II expression [125]. It was also observed by Wu et al. that AMPK inhibition led to an increase in inflammatory responses, inhibiting autophagy [123,126,127]. Additionally, ATG16L1 deficiency was shown to impair the conjugation of LC3-I to LC3-II, leading to increased IL-1β and IL-18 production in macrophages [65] (Table 3). Collectively, these findings underscore the regulatory role of autophagy in inflammation and pyroptotic cell death in INR (Figure 5). Thus, autophagy and pyroptosis emerge as essential mechanisms for understanding the deficiency of immunological recovery in PLWH, whose inability to maintain immune homeostasis contributes to disease progression toward AIDS.

## 8. Therapeutic Strategies and Perspectives

As described above, HIV is a virus that persists in individuals despite the effectiveness of ART. Strategies to modulate autophagy for viral elimination have been explored, including the use of ingenol 3,20-dibenzoate (IDB) to induce LC3 expression in CD4+ T cells, which helps protect uninfected cells from HIV-induced cell death [128]. However, the virus’s high mutation rate, the establishment of stable latent reservoirs, and the cumulative toxicity of some antiretrovirals remain major barriers to complete eradication [129,130,131].

Another approach involves the Tat-BECLIN-1 fusion peptide, which induces autophagy by binding to the HIV Nef protein, facilitating the elimination of latently infected central memory CD4+ T cells (TCM) while preventing virologic rebound [132] Another promising technique under investigation involves the use of CRISPR/Cas9 technology to target the retroviral restriction factor TRIM5α. Studies in human and monkey cell lines have shown that this approach has the potential to induce autophagy through interaction with the proteins ULK1 and LC3, leading to the degradation of HIV [133,134].

Innovative strategies for the elimination of HIV-1 reservoirs are being investigated, including the modulation of autophagy combined with cell death pathways. Recent studies have shown that the use of BH3 mimetics (molecules that compete with anti-apoptotic proteins in their interaction with pro-apoptotic proteins) has proven effective in inducing cell death in reactivated reservoirs, representing a promising advancement in the field of HIV-1 eradication [135].

Similarly, treatment with necrostatin-1 (Nec-1), a RIPK1 inhibitor that blocks the necroptosis pathway, significantly attenuated HIV-induced cytopathic effects, inhibiting the formation of HIV-induced syncytia in CD4 T-cell lines [110]. Pyroptosis inhibitors, such as those for caspase-1, have also been investigated for their potential to mitigate CD4+ T-cell loss, reduce chronic inflammation, and improve immune function, thereby minimizing HIV-associated tissue damage [23]. Furthermore, iron chelators such as deferoxamine have shown the ability to reduce ferroptotic cell death by limiting iron availability and promoting autophagy induction [136]. Collectively, these findings underscore the therapeutic potential of autophagy modulation in the management of HIV infection and a promising approach for INRs.

## 9. Conclusions

This review shows the interplay between autophagy, HIV, and various forms of programmed cell death, highlighting the complexity of viral interactions with host cellular mechanisms that facilitate HIV persistence and propagation. Autophagy is a central regulator of immune homeostasis and cell death, directly influencing T-lymphocyte development activation in PLWH. However, HIV can impair autophagic activation through the action of specific viral proteins, promoting increased cell death by pyroptosis and contributing to lymphocyte dysregulation, which significantly compromises the immune response PLWH under ART. Additionally, some antiretrovirals used in ART regimens may further contribute to reduce autophagy process, exacerbating immunological impairment in these individuals. Particularly emphasizing pyroptosis, this review highlights the influence of autophagy on cell death pathways and its role in the context of INR. Targeting this pathway may offer new insights for the development of more effective interventions in HIV infection management.

## Figures and Tables

**Table 1:** Interaction between HIV proteins and the autophagy process in immune system cells.

HIV Protein	Interaction with Autophagy Process	References
Env	In CD4+ T cells, it induces the accumulation of BECLIN-1 and apoptosis.	[36,77,82]
Env	In dendric cells, it stimulates mTOR activation and autophagy inhibition.	[83]
Gag	Gag-p17 suppress autophagy, interacting with LC3-II in macrophages.	[71]
Nef	In macrophages, it inhibits the maturation steps of autophagy via BECLIN-1.	[71]
Env	In bystander T cells, Env induces autophagy and promotes autophagic T-cell death.	[77]
Vpr	In CD4+ T cells, Vpr reduces LC3-II and BECLIN-1 levels.	[13]
Vif	In CD4 + T cells, Vif interacts with LC3-II, resulting in the inhibition of autophagy.	[80,84]
Vpu	In CD4 + T cells, Vpu interacts with LC3-III, suppressing autophagosome formation.	[70]

**Table 2:** Interaction between antiretrovirals and autophagy.

Antiretrovirals	Interaction with Autophagy	References
TDF + FTC + DTG	Abnormal lysosomal function and impairs autophagosome maturation	[93]
TDF + FTC + DTG	Increases ROS by inhibiting autophagic flux	[94]
TDF + FTC + RAL	Inhibits autophagy, blocking autophagosome formation	[95]
ZDV and d4T	Inhibits autophagy contributing to adverse effects	[96,97]
ATV	Stimulates autophagy	[99]

**Table 3:** Relationship between autophagic and pyroptosis.

Autophagic Process	Interaction with Pyroptosis	References
Elimination of PAMPs and DAMPs	Reduction in cell death by pyroptosis	[117,118]
ATG7 knockout	Increased inflammasome activation and elevated levels of IL-1β and IL-18	[118]
Degradation of NLRP3	Reduction in inflammasome	[119]
ULK-1 and ATG9	Suppression of STING signal and inhibition of inflammation and pyroptosis	[120]
Increased expression of BECLIN-1 and LC3II	Inhibition of pyroptosis via GDSM	[121]
Decrease in ATG5	Increase in ROS production and activation of NLRP3	[123]
Decreased expression of BECLIN-1	Activation of NLRP3 and other inflammatory mediators (IL-1β, END-1, and MCP)	[124]
Increase in ULK1	Decrease in pyroptosis via NOD2-RIP2	[125]
ATG16L1 deficiency	Stimulation of production of IL-1β and IL-18	[65]

## Data Availability

Non-applicable.

## References

[1] Xiao P., Chen X., Chen Y., Fan W., Dong Z., Huang J., Zhang Y. (2023). CD4^+^ T Cell Count in HIV/TB Co-Infection and Co-Occurrence with HL: Case Report and Literature Review. Open Life Sci..

[2] Wan L.-Y., Huang H.-H., Zhen C., Chen S.-Y., Song B., Cao W.-J., Shen L.-L., Zhou M.-J., Zhang X.-C., Xu R. (2023). Distinct Inflammation-Related Proteins Associated with T Cell Immune Recovery during Chronic HIV-1 Infection. Emerg. Microbes Infect..

[3] UNIAIDS (2024). Act Sheet 2024—Latest Global and Regional HIV Statistics on the Status. https://www.unaids.org/sites/default/files/media_asset/UNAIDS_FactSheet_en.pdf.

[4] Cabrera-Rodríguez R., Pérez-Yanes S., Estévez-Herrera J., Márquez-Arce D., Cabrera C., Espert L., Blanco J., Valenzuela-Fernández A. (2021). The Interplay of HIV and Autophagy in Early Infection. Front. Microbiol..

[5] Ministério da Saude (2024). Manejo Da Infecção Pelo HIV Em Adultos. https://www.gov.br/aids/pt-br/central-de-conteudo/pcdts/pcdt_hiv_modulo_1_2024.pdf.

[6] Yang X., Su B., Zhang X., Liu Y., Wu H., Zhang T. (2020). Incomplete Immune Reconstitution in HIV/AIDS Patients on Antiretroviral Therapy: Challenges of Immunological Non-Responders. J. Leukoc. Biol..

[7] Yan L., Xu K., Xiao Q., Tuo L., Luo T., Wang S., Yang R., Zhang F., Yang X. (2023). Cellular and Molecular Insights into Incomplete Immune Recovery in HIV/AIDS Patients. Front. Immunol..

[8] Loucif H., Dagenais-Lussier X., Avizonis D., Choinière L., Beji C., Cassin L., Routy J.-P., Fritz J.H., Olagnier D., Van Grevenynghe J. (2022). Autophagy-Dependent Glutaminolysis Drives Superior IL21 Production in HIV-1-Specific CD4 T Cells. Autophagy.

[9] Li S., Xu B., Luo Y., Luo J., Huang S., Guo X. (2024). Autophagy and Apoptosis in Rabies Virus Replication. Cells.

[10] Lamsira H.K., Sabatini A., Ciolfi S., Ciccosanti F., Sacchi A., Piacentini M., Nardacci R. (2025). Autophagy and Programmed Cell Death Modalities Interplay in HIV Pathogenesis. Cells.

[11] Jiang G.-M., Tan Y., Wang H., Peng L., Chen H.-T., Meng X.-J., Li L.-L., Liu Y., Li W.-F., Shan H. (2019). The Relationship between Autophagy and the Immune System and Its Applications for Tumor Immunotherapy. Mol. Cancer.

[12] Campbell G.R., Spector S.A. (2021). Induction of Autophagy to Achieve a Human Immunodeficiency Virus Type 1 Cure. Cells.

[13] Alfaisal J., Machado A., Galais M., Robert-Hebmann V., Arnauné-Pelloquin L., Espert L., Biard-Piechaczyk M. (2019). HIV-1 Vpr Inhibits Autophagy during the Early Steps of Infection of CD4 T Cells. Biol. Cell.

[14] Pant A., Yao X., Lavedrine A., Viret C., Dockterman J., Chauhan S., Shi C.-S., Manjithaya R., Cadwell K., Kufer T.A. (2022). Interactions of Autophagy and the Immune System in Health and Diseases. Autophagy Rep..

[15] Liu S., Yao S., Yang H., Liu S., Wang Y. (2023). Autophagy: Regulator of Cell Death. Cell Death Dis..

[16] Xiao Q., Yan L., Han J., Yang S., Tang Y., Li Q., Lao X., Chen Z., Xiao J., Zhao H. (2022). Metabolism-Dependent Ferroptosis Promotes Mitochondrial Dysfunction and Inflammation in CD4+ T Lymphocytes in HIV-Infected Immune Non-Responders. eBioMedicine.

[17] Seo J., Seong D., Nam Y.W., Hwang C.H., Lee S.R., Lee C.-S., Jin Y., Lee H.-W., Oh D.-B., Vandenabeele P. (2020). Beclin 1 Functions as a Negative Modulator of MLKL Oligomerisation by Integrating into the Necrosome Complex. Cell Death Differ..

[18] Wu W., Wang X., Sun Y., Berleth N., Deitersen J., Schlütermann D., Stuhldreier F., Wallot-Hieke N., José Mendiburo M., Cox J. (2021). TNF-Induced Necroptosis Initiates Early Autophagy Events via RIPK3-Dependent AMPK Activation, but Inhibits Late Autophagy. Autophagy.

[19] Alvarez-Meythaler J.G., Garcia-Mayea Y., Mir C., Kondoh H., LLeonart M.E. (2020). Autophagy Takes Center Stage as a Possible Cancer Hallmark. Front. Oncol..

[20] Sorice M. (2022). Crosstalk of Autophagy and Apoptosis. Cells.

[21] Vidya Vijayan K.K., Karthigeyan K.P., Tripathi S.P., Hanna L.E. (2017). Pathophysiology of CD4+ T-Cell Depletion in HIV-1 and HIV-2 Infections. Front. Immunol..

[22] Doitsh G., Greene W.C. (2016). Dissecting How CD4 T Cells Are Lost During HIV Infection. Cell Host Microbe.

[23] Klute S., Sparrer K.M.J. (2024). Friends and Foes: The Ambivalent Role of Autophagy in HIV-1 Infection. Viruses.

[24] Chaves M.D.T. (2019). Autophagy in the pathogenesis of HIV infection, Universidade de Lisboa. http://hdl.handle.net/10451/43436.

[25] Dinkins C., Arko-Mensah J., Deretic V. (2010). Autophagy and HIV. Semin. Cell Dev. Biol..

[26] Rubio-Tomás T., Sotiriou A., Tavernarakis N. (2023). The Interplay between Selective Types of (Macro) Autophagy: Mitophagy and Xenophagy. International Review of Cell and Molecular Biology.

[27] Silva H.R.d., Carvalho L.Q.C., Lira M.S., Oliveira J.P.T.d., Bringel L.A.F., Pinheiro Neto J.C., Miranda C.C.d.S., Salazar V.A.C., Costa R.H.F., Abreu H.M. (2021). Impact of the Autophagic Process on Antitumor Treatment in Pregnant Women. Rev. De. Casos E Consult..

[28] Chen T., Tu S., Ding L., Jin M., Chen H., Zhou H. (2023). The Role of Autophagy in Viral Infections. J. Biomed. Sci..

[29] Almansa-Gómez S., Prieto-Ruiz F., Cansado J., Madrid M. (2023). Autophagy Modulation as a Potential Therapeutic Strategy in Osteosarcoma: Current Insights and Future Perspectives. IJMS.

[30] Pedreño-López S., García E., Guerrero D., Gómez-Mora E., Mateu L.M., Pérez F.O., Senserrich J., Clotet B., Cabrera C. (2023). Author Correction: Modulation of the Autophagic Pathway Inhibits HIV-1 Infection in Human Lymphoid Tissue Cultured Ex Vivo. Sci. Rep..

[31] Giansanti M., Theinert T., Boeing S.K., Haas D., Schlegel P.-G., Vacca P., Nazio F., Caruana I. (2023). Exploiting Autophagy Balance in T and NK Cells as a New Strategy to Implement Adoptive Cell Therapies. Mol. Cancer.

[32] Yamamoto H., Zhang S., Mizushima N. (2023). Autophagy Genes in Biology and Disease. Nat. Rev. Genet..

[33] Vargas J.N.S., Hamasaki M., Kawabata T., Youle R.J., Yoshimori T. (2023). The Mechanisms and Roles of Selective Autophagy in Mammals. Nat. Rev. Mol. Cell Biol..

[34] Klapan K., Simon D., Karaulov A., Gomzikova M., Rizvanov A., Yousefi S., Simon H.-U. (2022). Autophagy and Skin Diseases. Front. Pharmacol..

[35] Santovito D., Steffens S., Barachini S., Madonna R. (2023). Autophagy, Innate Immunity, and Cardiac Disease. Front. Cell Dev. Biol..

[36] Nardacci R., Amendola A., Ciccosanti F., Corazzari M., Esposito V., Vlassi C., Taibi C., Fimia G.M., Del Nonno F., Ippolito G. (2014). Autophagy Plays an Important Role in the Containment of HIV-1 in Nonprogressor-Infected Patients. Autophagy.

[37] Jacquin E., Apetoh L. (2018). Cell-Intrinsic Roles for Autophagy in Modulating CD4 T Cell Functions. Front. Immunol..

[38] Xie Z., Klionsky D.J. (2007). Autophagosome Formation: Core Machinery and Adaptations. Nat. Cell Biol..

[39] Backer J.M. (2008). The Regulation and Function of Class III PI3Ks: Novel Roles for Vps34. Biochem. J..

[40] Lee Y., Tuan N.M., Lee G.J., Kim B., Park J.H., Lee C.H. (2024). Regulatory Mechanisms Governing the Autophagy-Initiating VPS34 Complex and Its Inhibitors. Biomol. Ther..

[41] Glick D., Barth S., Macleod K.F. (2010). Autophagy: Cellular and Molecular Mechanisms. J. Pathol..

[42] Barth S., Glick D., Macleod K.F. (2010). Autophagy: Assays and Artifacts. J. Pathol..

[43] Iriondo M.N., Etxaniz A., Varela Y.R., Ballesteros U., Lázaro M., Valle M., Fracchiolla D., Martens S., Montes L.R., Goñi F.M. (2023). Effect of ATG12–ATG5-ATG16L1 Autophagy E3-like Complex on the Ability of LC3/GABARAP Proteins to Induce Vesicle Tethering and Fusion. Cell. Mol. Life Sci..

[44] Ballesteros U., Iriondo M.N., Varela Y.R., Goñi F.M., Alonso A., Montes L.R., Etxaniz A. (2024). The N-Terminal Region of the ATG8 Autophagy Protein LC3C Is Essential for Its Membrane Fusion Properties. Int. J. Biol. Macromol..

[45] Johansen T., Lamark T. (2020). Selective Autophagy: ATG8 Family Proteins, LIR Motifs and Cargo Receptors. J. Mol. Biol..

[46] He H., Dang Y., Dai F., Guo Z., Wu J., She X., Pei Y., Chen Y., Ling W., Wu C. (2003). Post-Translational Modifications of Three Members of the Human MAP1LC3 Family and Detection of a Novel Type of Modification for MAP1LC3B. J. Biol. Chem..

[47] Wu J., Dang Y., Su W., Liu C., Ma H., Shan Y., Pei Y., Wan B., Guo J., Yu L. (2006). Molecular Cloning and Characterization of Rat LC3A and LC3B—Two Novel Markers of Autophagosome. Biochem. Biophys. Res. Commun..

[48] Bai H., Inoue J., Kawano T., Inazawa J. (2012). A Transcriptional Variant of the LC3A Gene Is Involved in Autophagy and Frequently Inactivated in Human Cancers. Oncogene.

[49] Koukourakis M.I., Kalamida D., Giatromanolaki A., Zois C.E., Sivridis E., Pouliliou S., Mitrakas A., Gatter K.C., Harris A.L. (2015). Autophagosome Proteins LC3A, LC3B and LC3C Have Distinct Subcellular Distribution Kinetics and Expression in Cancer Cell Lines. PLoS ONE.

[50] Yoshii S.R., Mizushima N. (2017). Monitoring and Measuring Autophagy. IJMS.

[51] Popelka H., Klionsky D.J. (2024). When an Underdog Becomes a Major Player: The Role of Protein Structural Disorder in the Atg8 Conjugation System. Autophagy.

[52] Ganley I.G., Lam D.H., Wang J., Ding X., Chen S., Jiang X. (2009). ULK1·ATG13·FIP200 Complex Mediates mTOR Signaling and Is Essential for Autophagy. J. Biol. Chem..

[53] Saxton R.A., Sabatini D.M. (2017). mTOR Signaling in Growth, Metabolism, and Disease. Cell.

[54] Geng J., Klionsky D.J. (2008). The Atg8 and Atg12 Ubiquitin-like Conjugation Systems in Macroautophagy. EMBO Rep..

[55] Lőrincz P., Juhász G. (2020). Autophagosome-Lysosome Fusion. J. Mol. Biol..

[56] Debnath J., Gammoh N., Ryan K.M. (2023). Autophagy and Autophagy-Related Pathways in Cancer. Nat. Rev. Mol. Cell Biol..

[57] Parzych K.R., Klionsky D.J. (2014). An Overview of Autophagy: Morphology, Mechanism, and Regulation. Antioxid. Redox Signal..

[58] Arbogast F., Gros F. (2018). Lymphocyte Autophagy in Homeostasis, Activation, and Inflammatory Diseases. Front. Immunol..

[59] Leymarie O., Lepont L., Berlioz-Torrent C. (2017). Canonical and Non-Canonical Autophagy in HIV-1 Replication Cycle. Viruses.

[60] Gómez-Virgilio L., Silva-Lucero M.-C., Flores-Morelos D.-S., Gallardo-Nieto J., Lopez-Toledo G., Abarca-Fernandez A.-M., Zacapala-Gómez A.-E., Luna-Muñoz J., Montiel-Sosa F., Soto-Rojas L.O. (2022). Autophagy: A Key Regulator of Homeostasis and Disease: An Overview of Molecular Mechanisms and Modulators. Cells.

[61] Deng S., Liu J., Wu X., Lu W. (2020). Golgi Apparatus: A Potential Therapeutic Target for Autophagy-Associated Neurological Diseases. Front. Cell Dev. Biol..

[62] Tian X., Teng J., Chen J. (2021). New Insights Regarding SNARE Proteins in Autophagosome-Lysosome Fusion. Autophagy.

[63] Wu M.-Y., Lu J.-H. (2019). Autophagy and Macrophage Functions: Inflammatory Response and Phagocytosis. Cells.

[64] Sanjuan M.A., Dillon C.P., Tait S.W.G., Moshiach S., Dorsey F., Connell S., Komatsu M., Tanaka K., Cleveland J.L., Withoff S. (2007). Toll-like Receptor Signalling in Macrophages Links the Autophagy Pathway to Phagocytosis. Nature.

[65] Saitoh T., Fujita N., Jang M.H., Uematsu S., Yang B.-G., Satoh T., Omori H., Noda T., Yamamoto N., Komatsu M. (2008). Loss of the Autophagy Protein Atg16L1 Enhances Endotoxin-Induced IL-1β Production. Nature.

[66] Romao S., Gasser N., Becker A.C., Guhl B., Bajagic M., Vanoaica D., Ziegler U., Roesler J., Dengjel J., Reichenbach J. (2013). Autophagy Proteins Stabilize Pathogen-Containing Phagosomes for Prolonged MHC II Antigen Processing. J. Cell Biol..

[67] Cooney R., Baker J., Brain O., Danis B., Pichulik T., Allan P., Ferguson D.J.P., Campbell B.J., Jewell D., Simmons A. (2010). NOD2 Stimulation Induces Autophagy in Dendritic Cells Influencing Bacterial Handling and Antigen Presentation. Nat. Med..

[68] Jagannath C., Lindsey D.R., Dhandayuthapani S., Xu Y., Hunter R.L., Eissa N.T. (2009). Autophagy Enhances the Efficacy of BCG Vaccine by Increasing Peptide Presentation in Mouse Dendritic Cells. Nat. Med..

[69] Rodriguez M., Lapierre J., Ojha C.R., Pawitwar S., Karuppan M.K.M., Kashanchi F., El-Hage N. (2019). Morphine Counteracts the Antiviral Effect of Antiretroviral Drugs and Causes Upregulation of P62/SQSTM1 and Histone-Modifying Enzymes in HIV-Infected Astrocytes. J. Neurovirol..

[70] Madjo U., Leymarie O., Frémont S., Kuster A., Nehlich M., Gallois-Montbrun S., Janvier K., Berlioz-Torrent C. (2016). LC3C Contributes to Vpu-Mediated Antagonism of BST2/Tetherin Restriction on HIV-1 Release through a Non-Canonical Autophagy Pathway. Cell Rep..

[71] Kyei G.B., Dinkins C., Davis A.S., Roberts E., Singh S.B., Dong C., Wu L., Kominami E., Ueno T., Yamamoto A. (2009). Autophagy Pathway Intersects with HIV-1 Biosynthesis and Regulates Viral Yields in Macrophages. J. Cell Biol..

[72] Zhang M.-Q., Li J.-R., Yang L., Peng Z.-G., Wu S., Zhang J.-P. (2024). ATG10S Promotes IFNL1 Expression and Autophagic Degradation of Multiple Viral Proteins Mediated by IFNL1. Autophagy.

[73] Zhang M., Li L., Wu L., Zhang J. (2022). Isarubrolone C Promotes Autophagic Degradation of Virus Proteins via Activating ATG10S in HepG2 Cells. J. Nat. Prod..

[74] Judith D., Berlioz-Torrent C. (2024). The Autophagy-Related Protein ATG5 Is a Central Mediator of a Non-Canonical Autophagy Pathway Hijacked by HIV-1 to Weaken the Host’s Response to Infection. Autophagy.

[75] Espert L., Varbanov M., Robert-Hebmann V., Sagnier S., Robbins I., Sanchez F., Lafont V., Biard-Piechaczyk M. (2009). Differential Role of Autophagy in CD4 T Cells and Macrophages during X4 and R5 HIV-1 Infection. PLoS ONE.

[76] Killian M. (2012). Dual Role of Autophagy in HIV-1 Replication and Pathogenesis. AIDS Res. Ther..

[77] Espert L. (2006). Autophagy Is Involved in T Cell Death after Binding of HIV-1 Envelope Proteins to CXCR4. J. Clin. Investig..

[78] Casado C., Pernas M., Sandonis V., Alvaro-Cifuentes T., Olivares I., Fuentes R., Martínez-Prats L., Grau E., Ruiz L., Delgado R. (2013). Identification of a Cluster of HIV-1 Controllers Infected with Low Replicating Viruses. PLoS ONE.

[79] Casado C., Marrero-Hernández S., Márquez-Arce D., Pernas M., Marfil S., Borràs-Grañana F., Olivares I., Cabrera-Rodríguez R., Valera M.-S., De Armas-Rillo L. (2018). Viral Characteristics Associated with the Clinical Nonprogressor Phenotype Are Inherited by Viruses from a Cluster of HIV-1 Elite Controllers. mBio.

[80] Liu Z., Xiao Y., Torresilla C., Rassart É., Barbeau B. (2017). Implication of Different HIV-1 Genes in the Modulation of Autophagy. Viruses.

[81] Moreira D., Silvestre R., Cordeiro-da-Silva A., Estaquier J., Foretz M., Viollet B. (2016). AMP-Activated Protein Kinase As a Target For Pathogens: Friends Or Foes?. CDT.

[82] Perfettini J.-L., Castedo M., Roumier T., Andreau K., Nardacci R., Piacentini M., Kroemer G. (2005). Mechanisms of Apoptosis Induction by the HIV-1 Envelope. Cell Death Differ..

[83] Blanchet F.P., Moris A., Nikolic D.S., Lehmann M., Cardinaud S., Stalder R., Garcia E., Dinkins C., Leuba F., Wu L. (2010). Human Immunodeficiency Virus-1 Inhibition of Immunoamphisomes in Dendritic Cells Impairs Early Innate and Adaptive Immune Responses. Immunity.

[84] Borel S., Robert-Hebmann V., Alfaisal J., Jain A., Faure M., Espert L., Chaloin L., Paillart J.-C., Johansen T., Biard-Piechaczyk M. (2015). HIV-1 Viral Infectivity Factor Interacts with Microtubule-Associated Protein Light Chain 3 and Inhibits Autophagy. AIDS.

[85] Rosenberg E.S., Billingsley J.M., Caliendo A.M., Boswell S.L., Sax P.E., Kalams S.A., Walker B.D. (1997). Vigorous HIV-1-Specific CD4^+^ T Cell Responses Associated with Control of Viremia. Science.

[86] Addison M.M., Ellis G.I., Leslie G.J., Zawadzky N.B., Riley J.L., Hoxie J.A., Eisenlohr L.C. (2022). HIV-1–Infected CD4+ T Cells Present MHC Class II–Restricted Epitope via Endogenous Processing. J. Immunol..

[87] Guedes M.C.S., Lopes-Araujo H.F., Dos Santos K.F., Simões E., Carvalho-Silva W.H.V., Guimarães R.L. (2025). How to Properly Define Immunological Nonresponse to Antiretroviral Therapy in People Living with HIV? An Integrative Review. Front. Immunol..

[88] Carvalho-Silva W.H.V., Andrade-Santos J.L., Souto F.O., Coelho A.V.C., Crovella S., Guimarães R.L. (2020). Immunological Recovery Failure in cART-Treated HIV-Positive Patients Is Associated with Reduced Thymic Output and RTE CD4+ T Cell Death by Pyroptosis. J. Leukoc. Biol..

[89] Santos J.L.d.A. (2022). Fatores Do Hospedeiro Associados à Morte Celular Na Recuperação Imunológica de Indivíduos HIV-1 Positivos Submetidos à Terapia Antirretroviral.

[90] Corbeau P., Reynes J. (2011). Immune Reconstitution under Antiretroviral Therapy: The New Challenge in HIV-1 Infection. Blood.

[91] World Health Organization (2024). HIV Drug Resistance. https://www.who.int/teams/global-hiv-hepatitis-and-stis-programmes/hiv/treatment/hiv-drug-resistance.

[92] Chan P., Goh O., Kroon E., Colby D., Sacdalan C., Pinyakorn S., Prueksakaew P., Reiss P., Ananworanich J., Valcour V. (2020). Neuropsychiatric Outcomes before and after Switching to Dolutegravir-Based Therapy in an Acute HIV Cohort. AIDS Res. Ther..

[93] Tripathi A., Thangaraj A., Chivero E.T., Periyasamy P., Callen S., Burkovetskaya M.E., Guo M.-L., Buch S. (2019). Antiretroviral-Mediated Microglial Activation Involves Dysregulated Autophagy and Lysosomal Dysfunction. Cells.

[94] Tripathi A., Thangaraj A., Chivero E.T., Periyasamy P., Burkovetskaya M.E., Niu F., Guo M.-L., Buch S. (2020). N-Acetylcysteine Reverses Antiretroviral-Mediated Microglial Activation by Attenuating Autophagy-Lysosomal Dysfunction. Front. Neurol..

[95] Cheney L., Guzik H., Macaluso F.P., Macian F., Cuervo A.M., Berman J.W. (2020). HIV Nef and Antiretroviral Therapy Have an Inhibitory Effect on Autophagy in Human Astrocytes That May Contribute to HIV-Associated Neurocognitive Disorders. Cells.

[96] Stankov M.V., Panayotova-Dimitrova D., Leverkus M., Schmidt R.E., Behrens G.M.N. (2013). Thymidine Analogues Suppress Autophagy and Adipogenesis in Cultured Adipocytes. Antimicrob. Agents Chemother..

[97] Lin H., Stankov M.V., Hegermann J., Budida R., Panayotova-Dimitrova D., Schmidt R.E., Behrens G.M.N. (2019). Zidovudine-Mediated Autophagy Inhibition Enhances Mitochondrial Toxicity in Muscle Cells. Antimicrob. Agents Chemother..

[98] World Health Organization (2018). Updated Recommendations on First-Line and Second-Line Antiretroviral Regimens and Post-Exposure Prophylaxis and Recommendations on Early Infant Diagnosis of HIV: Interim Guidelines: Supplement to the 2016 Consolidated Guidelines on the Use of Antiretroviral Drugs for Treating and Preventing HIV Infection. https://www.who.int/publications/i/item/WHO-CDS-HIV-18.51.

[99] Gibellini L., De Biasi S., Pinti M., Nasi M., Riccio M., Carnevale G., Cavallini G.M., Sala De Oyanguren F.J., O’Connor J.E., Mussini C. (2012). The Protease Inhibitor Atazanavir Triggers Autophagy and Mitophagy in Human Preadipocytes. AIDS.

[100] Patties I., Kortmann R.-D., Menzel F., Glasow A. (2016). Enhanced Inhibition of Clonogenic Survival of Human Medulloblastoma Cells by Multimodal Treatment with Ionizing Irradiation, Epigenetic Modifiers, and Differentiation-Inducing Drugs. J. Exp. Clin. Cancer Res..

[101] Bellisai C., Sciamanna I., Rovella P., Giovannini D., Baranzini M., Pugliese G.M., Zeya Ansari M.S., Milite C., Sinibaldi-Vallebona P., Cirilli R. (2020). Reverse Transcriptase Inhibitors Promote the Remodelling of Nuclear Architecture and Induce Autophagy in Prostate Cancer Cells. Cancer Lett..

[102] La Rosa F., Saresella M., Marventano I., Piancone F., Ripamonti E., Al-Daghri N., Bazzini C., Zoia C.P., Conti E., Ferrarese C. (2019). Stavudine Reduces NLRP3 Inflammasome Activation and Modulates Amyloid-β Autophagy. JAD.

[103] Sagnier S., Daussy C.F., Borel S., Robert-Hebmann V., Faure M., Blanchet F.P., Beaumelle B., Biard-Piechaczyk M., Espert L. (2015). Autophagy Restricts HIV-1 Infection by Selectively Degrading Tat in CD4^+^ T Lymphocytes. J. Virol..

[104] Arsov I., Adebayo A., Kucerova-Levisohn M., Haye J., MacNeil M., Papavasiliou F.N., Yue Z., Ortiz B.D. (2011). A Role for Autophagic Protein Beclin 1 Early in Lymphocyte Development. J. Immunol..

[105] Pua H.H., Dzhagalov I., Chuck M., Mizushima N., He Y.-W. (2007). A Critical Role for the Autophagy Gene Atg5 in T Cell Survival and Proliferation. J. Exp. Med..

[106] Mortensen M., Watson A.S., Simon A.K. (2011). Lack of Autophagy in the Hematopoietic System Leads to Loss of Hematopoietic Stem Cell Function and Dysregulated Myeloid Proliferation. Autophagy.

[107] Coulon P.-G., Richetta C., Rouers A., Blanchet F.P., Urrutia A., Guerbois M., Piguet V., Theodorou I., Bet A., Schwartz O. (2016). HIV-Infected Dendritic Cells Present Endogenous MHC Class II–Restricted Antigens to HIV-Specific CD4+ T Cells. J. Immunol..

[108] Cho Y., Challa S., Moquin D., Genga R., Ray T.D., Guildford M., Chan F.K.-M. (2009). Phosphorylation-Driven Assembly of the RIP1-RIP3 Complex Regulates Programmed Necrosis and Virus-Induced Inflammation. Cell.

[109] Zhou Z., Han V., Han J. (2012). New Components of the Necroptotic Pathway. Protein Cell.

[110] Pan T., Wu S., He X., Luo H., Zhang Y., Fan M., Geng G., Ruiz V.C., Zhang J., Mills L. (2014). Necroptosis Takes Place in Human Immunodeficiency Virus Type-1 (HIV-1)-Infected CD4+ T Lymphocytes. PLoS ONE.

[111] Rojas-Rivera D., Beltrán S., Muñoz-Carvajal F., Ahumada-Montalva P., Abarzúa L., Gomez L., Hernandez F., Bergmann C.A., Labrador L., Calegaro-Nassif M. (2024). The Autophagy Protein RUBCNL/PACER Represses RIPK1 Kinase-Dependent Apoptosis and Necroptosis. Autophagy.

[112] Ciechomska I.A. (2018). Rola Autofagii w Komórkach Nowotworowych: Charakterystyka Wzajemnych Zależności Pomiędzy Procesami Autofagii i Apoptozy; Modulacja Autofagii Jako Nowa Strategia Terapeutyczna w Leczeniu Glejaków. Postep. Biochem..

[113] Kang R., Zeh H.J., Lotze M.T., Tang D. (2011). The Beclin 1 Network Regulates Autophagy and Apoptosis. Cell Death Differ..

[114] Takahashi Y., Meyerkord C.L., Wang H.-G. (2009). Bif-1/Endophilin B1: A Candidate for Crescent Driving Force in Autophagy. Cell Death Differ..

[115] Gu W., Wan D., Qian Q., Yi B., He Z., Gu Y., Wang L., He S. (2014). Ambra1 Is an Essential Regulator of Autophagy and Apoptosis in SW620 Cells: Pro-Survival Role of Ambra1. PLoS ONE.

[116] Sun W., He L., Liang L., Liu S., Luo J., Lv M., Cai Z. (2022). Ambra1 Regulates Apoptosis and Chemosensitivity in Breast Cancer Cells through the Akt-FoxO1-Bim Pathway. Apoptosis.

[117] Man S.M., Karki R., Kanneganti T. (2017). Molecular Mechanisms and Functions of Pyroptosis, Inflammatory Caspases and Inflam-masomes in Infectious Diseases. Immunol. Rev..

[118] Guo R., Wang H., Cui N. (2021). Autophagy Regulation on Pyroptosis: Mechanism and Medical Implication in Sepsis. Mediat. Inflamm..

[119] Chen Y., Luo Y., Liu Y., Qiu X., Luo D., Liu A. (2025). Mediation of Macrophage M1 Polarization Dynamics Change by Ubiquitin-Autophagy-Pathway Regulated NLRP3 Inflammasomes in PD-1 Inhibitor-Related Myocardial Inflammatory Injury. Inflamm. Res..

[120] Gui X., Yang H., Li T., Tan X., Shi P., Li M., Du F., Chen Z.J. (2019). Autophagy Induction via STING Trafficking Is a Primordial Function of the cGAS Pathway. Nature.

[121] Hua T., Yang M., Song H., Kong E., Deng M., Li Y., Li J., Liu Z., Fu H., Wang Y. (2022). Huc-MSCs-Derived Exosomes Attenuate Inflammatory Pain by Regulating Microglia Pyroptosis and Autophagy via the miR-146a-5p/TRAF6 Axis. J. Nanobiotechnol..

[122] Wu J., Li X., Zhu G., Zhang Y., He M., Zhang J. (2016). The Role of Resveratrol-Induced Mitophagy/Autophagy in Peritoneal Mesothelial Cells Inflammatory Injury via NLRP3 Inflammasome Activation Triggered by Mitochondrial ROS. Exp. Cell Res..

[123] Elrashidy R.A., Mohamad H.E., Aal S.M.A., Mohamed S.R., Tolba S.M., Mahmoud Y.K. (2025). Repurposing Secukinumab and Dapagliflozin as Candidate Therapies to Mitigate the Renal Toxicity of Sunitinib in Rats Through Suppressing IL-17-Mediated Pyroptosis and Promoting Autophagy. J. Biochem. Amp; Mol. Tox.

[124] Wang G., Zhang C., Jiang F., Zhao M., Xie S., Liu X. (2022). NOD2-RIP2 Signaling Alleviates Microglial ROS Damage and Pyroptosis via ULK1-Mediated Autophagy during Streptococcus Pneumonia Infection. Neurosci. Lett..

[125] Luo T., Jia X., Feng W., Wang J., Xie F., Kong L., Wang X., Lian R., Liu X., Chu Y. (2023). Bergapten Inhibits NLRP3 Inflammasome Activation and Pyroptosis via Promoting Mitophagy. Acta Pharmacol. Sin..

[126] Wang Y., Viollet B., Terkeltaub R., Liu-Bryan R. (2016). AMP-Activated Protein Kinase Suppresses Urate Crystal-Induced Inflammation and Transduces Colchicine Effects in Macrophages. Ann. Rheum. Dis..

[127] McWherter C., Choi Y.-J., Serrano R.L., Mahata S.K., Terkeltaub R., Liu-Bryan R. (2018). Arhalofenate Acid Inhibits Monosodium Urate Crystal-Induced Inflammatory Responses through Activation of AMP-Activated Protein Kinase (AMPK) Signaling. Arthritis Res. Ther..

[128] Li M., Liu W., Bauch T., Graviss E.A., Arduino R.C., Kimata J.T., Chen M., Wang J. (2020). Clearance of HIV Infection by Selective Elimination of Host Cells Capable of Producing HIV. Nat. Commun..

[129] Decloedt E.H., Rosenkranz B., Maartens G., Joska J. (2015). Central Nervous System Penetration of Antiretroviral Drugs: Pharmacokinetic, Pharmacodynamic and Pharmacogenomic Considerations. Clin. Pharmacokinet..

[130] Huang H., Kong W., Jean M., Fiches G., Zhou D., Hayashi T., Que J., Santoso N., Zhu J. (2019). A CRISPR/Cas9 Screen Identifies the Histone Demethylase MINA53 as a Novel HIV-1 Latency-Promoting Gene (LPG). Nucleic Acids Res..

[131] Eisele E., Siliciano R.F. (2012). Redefining the Viral Reservoirs That Prevent HIV-1 Eradication. Immunity.

[132] Zhang G., Luk B.T., Wei X., Campbell G.R., Fang R.H., Zhang L., Spector S.A. (2019). Selective Cell Death of Latently HIV-Infected CD4+ T Cells Mediated by Autosis Inducing Nanopeptides. Cell Death Dis..

[133] Keown J.R., Black M.M., Ferron A., Yap M., Barnett M.J., Pearce F.G., Stoye J.P., Goldstone D.C. (2018). A Helical LC3-Interacting Region Mediates the Interaction between the Retroviral Restriction Factor Trim5α and Mammalian Autophagy-Related ATG8 Proteins. J. Biol. Chem..

[134] Cloherty A.P.M., Rader A.G., Compeer B., Ribeiro C.M.S. (2021). Human TRIM5α: Autophagy Connects Cell-Intrinsic HIV-1 Restriction and Innate Immune Sensor Functioning. Viruses.

[135] Chen M., Li M., Budai M.M., Rice A.P., Kimata J.T., Mohan M., Wang J. (2022). Clearance of HIV-1 or SIV Reservoirs by Promotion of Apoptosis and Inhibition of Autophagy: Targeting Intracellular Molecules in Cure-Directed Strategies. J. Leukoc. Biol..

[136] Zhang F., Yan Y., Cai Y., Liang Q., Liu Y., Peng B., Xu Z., Liu W. (2023). Current Insights into the Functional Roles of Ferroptosis in Musculoskeletal Diseases and Therapeutic Implications. Front. Cell Dev. Biol..

